# LAG3: The Biological Processes That Motivate Targeting This Immune Checkpoint Molecule in Human Cancer

**DOI:** 10.3390/cancers11081213

**Published:** 2019-08-20

**Authors:** Cinzia Solinas, Edoardo Migliori, Pushpamali De Silva, Karen Willard-Gallo

**Affiliations:** 1 Molecular Immunology Unit, Institut Jules Bordet, Université Libre de Bruxelles, 1000 Brussels, Belgium; 2 Azienda Unità Sanitaria Locale Valle d’Aosta, Regional Hospital of Aosta, 11100 Aosta, Italy; 3 Columbia University Medical Center, Columbia Center for Translational Immunology, NY 10032, USA

**Keywords:** LAG3, immune checkpoint, immunotherapy, breast cancer

## Abstract

The programmed cell death 1 (PD-1) pathway is an important regulator of immune responses in peripheral tissues, including abnormal situations such as the tumor microenvironment. This pathway is currently the principal target for immunotherapeutic compounds designed to block immune checkpoint pathways, with these drugs improving clinical outcomes in a number of solid and hematological tumors. Medical oncology is experiencing an immune revolution that has scientists and clinicians looking at alternative, non-redundant inhibitory pathways also involved in regulating immune responses in cancer. A variety of targets have emerged for combinatorial approaches in immune checkpoint blockade. The main purpose of this narrative review is to summarize the biological role of lymphocyte activation gene 3 (LAG3), an emerging targetable inhibitory immune checkpoint molecule. We briefly discuss its role in infection, autoimmune disease and cancer, with a more detailed analysis of current data on LAG3 expression in breast cancer. Current clinical trials testing soluble LAG3 immunoglobulin and LAG3 antagonists are also presented in this work.

## 1. Introduction

Improved clinical outcomes have been achieved for a number of solid and hematological diseases treated with immune checkpoint blockade (ICB) targeting cytotoxic T lymphocyte associated protein (CTLA)-4 and programmed cell death 1 (PD-1) or its ligand PD-L1 [1,2,3,4,5,6,7,8,9,10,11]. Nevertheless, a large proportion of ICB-treated cancer patients still do not benefit from these drugs. Thus, while initial ICB targets have led to an immunological resurgence in oncology, this lack of widespread clinical benefit together with the occurrence of immune related adverse events (irAEs), principally due to the onset of autoimmune reactions [12,13,14,15,16,17], have focused attention on alternative inhibitory immune checkpoint molecules, including lymphocyte activation gene 3 (LAG3, CD223), T cell immunoglobulin mucin 3 (TIM3) [18], adenosine A2A receptor (A2AR) [19], indoleamine-pyrrole 2,3-dioxygenase (IDO) [20], T cell immunoreceptor with Immunoglobulin and ITIM domains (TIGIT) [21], CD96 [22] and many others [23]. 

LAG3 is the third inhibitory receptor pathway to be targeted in the clinic. LAG3 functions to control excessive activation following persistent antigen (Ag) exposure in an effort to prevent the onset of autoimmunity [24,25]; however, it can also contribute to a state of T cell dysfunction in the tumor microenvironment (TME) [24,26,27]. Dysfunctional T cells are characterized by impaired proliferation and cytokine production that distinguishes their inability to exert effector functions despite previous Ag encounters. Ineffective T cells have been detected in chronic inflammatory settings, including autoimmune diseases and tumors (i.e., tumor infiltrating lymphocytes or TIL). Various drugs targeting LAG3 are now available in the clinic with many more under development [28]. The aim of this review is to provide an overview of LAG3 biology under physiological and pathological conditions associated with chronic inflammatory microenvironments, such as that observed in various tumors including breast cancer (BC). Further, we include an overview of ongoing early phase clinical trials targeting LAG3 in oncology.

## 2. LAG3 Biological Activities and Expression

**Figure 1:**
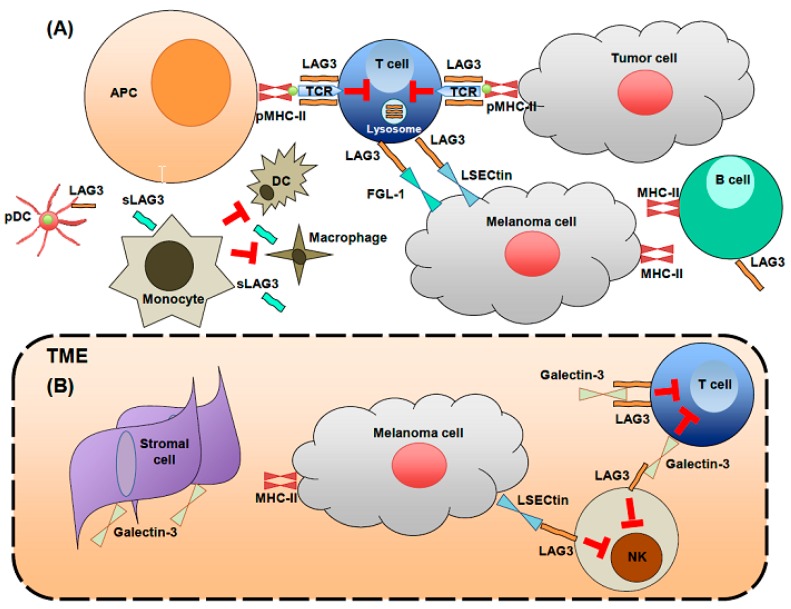
LAG3 biology on immune cells (**A**) and in the tumor microenvironment (TME) (**B**). (**A**). LAG3 is expressed on CD4^+^ (Th) and CD8^+^ (CTL) T cells, plasmacytoid dendritic cells (pDC) and NK cells in the TME (**B**). Its principle ligands include: MHC-II expressed on antigen presenting cells (APC) and tumor cells, LSECtin expressed on melanoma cells and galectin-3 expressed on some T cells and stromal cells in the TME (**B**). In its soluble form, LAG3 (sLAG3) impairs monocyte differentiation to dendritic cells (DC) and macrophages. In the TME, interactions mediated by LAG3 and its ligands are inhibitory (**B**).

The LAG3 receptor is expressed on: 1) Activated human CD4^+^ (helper = Th) and CD8^+^ (cytotoxic = CTL) T cells where it is detectable within 24 h following in vitro stimulation [29]; 2) a subset of natural killer (NK) cells and invariant NK T cells [28,29]; and 3) murine plasmacytoid dendritic cells (pDC) where it was shown to be constitutively expressed [30], although this latter finding has not been confirmed. In addition, LAG3 expression was detected on B cells [31,32,33] and neurons [34], but these data also need validation. Apart from its membrane expression, LAG3 can also be stored in lysosomes, which facilitates its rapid appearance on the cell surface following T cell activation [35]. LAG3 principally interacts with major histocompatibility complex class II (MHC-II) molecules as its ligand, which is expressed on the surface of Ag presenting cells (APCs) and tumor cells [36,37] (Figure 1). A recent study has provided further insight by showing that LAG3^+^ CD4^+^ T cells selectively interact with stably expressed peptide-MHC-II complexes [38].

Activated effector CD4^+^ T cells, in particular regulatory T cells (Tregs) including both activated natural (nTregs) and induced CD4^+^ FoxP3^+^ Tregs (iTregs), express LAG3 [39] with their suppressive functions inhibited when it is blocked. Further, ectopic expression of LAG3 confers suppressive activity to non-Treg CD4^+^ T cells [38]. LAG3 inhibits CD4^+^ T cell activation via its conformational dependent recognition of stable peptide-MHC-II complexes [38]. A recent study found that LAG3 can also play a role in maintaining the quiescence of naïve CD4^+^ T cells [40]. LAG3 expression has been detected on highly immunosuppressive CD4^+^ FoxP3^−^ IL10-secreting type 1 regulatory (Tr1) T cells, which have been identified in both humans and mice by dual expression of LAG3 and CD49b [41]. Tr1 cells are thought to play a role in promoting and maintaining tolerance in the periphery where they are induced. In cancer patients, LAG3 is preferentially expressed on Treg TIL in the TME relative to those in the periphery [41]. LAG3 can also inhibit APC (DC) functions by blocking their maturation when bound to LAG3^+^ Tregs [42].

**Figure 2:**
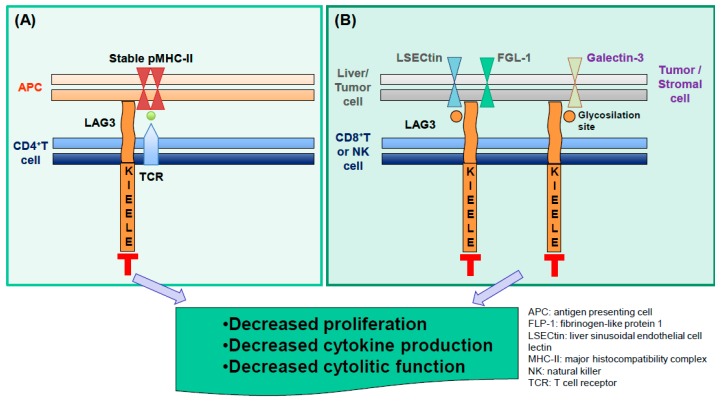
Molecular mechanisms of LAG3 function. LAG3 can be expressed on CD4^+^ (**A**), CD8^+^ T and NK cells (**B**). Its interaction with its ligands (stable pMHC-II complexes; LSECtin; FGL-1; galectin-3) expressed on different cells (immune, stromal, liver and tumor cells), generates an inhibition of CD4^+^, CD8^+^ and NK T cell proliferation, cytokine production and cytolitic function. These effects are mediated by the cytoplasmic motif of the LAG3 receptor, which is named KIEELE.

LAG3 expression on CD8^+^ T and NK cells symbolizes a dysfunctional profile [43], although its effect on NK cells requires further investigation because blocking this pathway did not modify human NK cytotoxicity [43,44,45,46,47,48]. Because LAG3 impacts functionality, it has been suggested that it can bind ligands other than MHC-II, the latter constitutively expressed on APC and B cells, inducibly expressed on CD4^+^ T cells and other immune (neutrophils and eosinophils) and stromal cells (fibroblasts) and also expressed on many types of tumor cells [49,50] (Figure 1 and Figure 2).

Potential LAG3 ligands include: 1) Liver sinusoidal endothelial cell lectin (LSECtin), a member of the DC-SIGN family, which is expressed in the liver and on melanoma cells, where it functions to inhibit anti-tumor CD8^+^ T cell [51] and NK responses; 2) galectin-3, which is expressed by stromal cells [52,53] and CD8^+^ T cells in mice [54], which can bind LAG3 under conditions of heavy glycosylation [44]; 3) α-synuclein in neurons [55]; and the recently demonstrated fibrinogen-like protein 1, released in the liver (at low levels) and by tumor cells [34] ( Figure 1; Figure 2).

LAG3 also has a soluble form (sLAG3) released by shedding at the cell surface that provides an additional layer of control and immune regulation in the periphery or TME. sLAG3 is thought to impair monocyte differentiation into macrophages or DC, producing APC that ultimately have reduced immunostimulatory capacities [56]. It has also been studied as a circulating biomarker in BC patients with hormone receptor (HR)-positive metastatic disease, where detectable serum sLAG3 at diagnosis was associated with a survival advantage [57] with similar findings in a recent study of gastric cancer [58]. These data advocate further investigation of sLAG3 as a prognostic or predictive biomarker for LAG3-targeted and other cancer therapies.

## 3. LAG3 Expression at Sites Characterized by Chronic Immune Activation

LAG3 plays a protective role in autoimmune diseases by dampening CD4^+^ T cell responses through MHC-II engagement and inhibiting effector T cell responses by promoting Treg and Tr1 suppression. This checkpoint regulation, designed to curb excessive immune activities [59], was clearly demonstrated by its synergistic activity with PD-1 to prevent autoimmunity in mice [60]. In chronic infection models, LAG3 expression was directly correlated with infection severity [61]. Furthermore, it was co-expressed with PD-1 on dysfunctional virus-specific CD8^+^ T cells and parasite-specific CD4^+^ T cells [62]. Notably, while LAG3 blockade alone had little effect [61], the synergistic effect of adding PD-L1 blockade improved CD8^+^ [61] and CD4^+^ [62] T cell responses. The role LAG3 plays in the dysfunction of T cell TIL is similar to that observed during chronic viral infections. Following sustained Ag exposure activated CD4^+^ and CD8^+^ T cells upregulate multiple inhibitory receptors, and are characterized by impaired proliferation, cytokine secretion (IFNγ and TNFα) and cytolytic activity [59], as poorly functional lymphocytes.

LAG3 and PD-1 co-expression has been used to identify dysfunctional CD8^+^ T cells in human tumors with their co-blockade in ovarian cancer improving proliferation and cytokine production by tumor-Ag-specific CD8^+^ T cells [63]. Further, in metastatic ovarian cancer it was shown that LAG3 activity as a single agent was limited by the expression of other inhibitory immune checkpoint molecules (i.e. PD-1 and CTLA-4) [64]. In colorectal carcinoma, LAG3 was expressed at higher levels in microsatellite unstable compared with microsatellite stable tumors [65]. LAG3 was also shown to be highly expressed on Tregs in the peripheral blood, metastatic lymph nodes and tumor tissues from melanoma and colorectal carcinoma [66], head and neck squamous cell carcinoma and non-small cell lung cancer patients [67,68]. These LAG3^+^ Tregs have an activated, immunosuppressive phenotype and produce high levels of IL-10 and TGFβ1 [66].

## 4. LAG3 in Human Breast Cancer

A recent study by our group examined a series of targetable inhibitory immune checkpoint molecules in primary BC, finding that PD-1 and CTLA-4 were consistently highly expressed on specific T cell subpopulations [69]. We detected LAG3 expression on a small proportion of CD4^+^ and CD8^+^ TIL but only rarely on stromal and non-lymphoid cells in the TME. Remarkably, all tumors that expressed LAG3^+^ also contained PD-1^+^ CD4^+^ and/or CD8^+^ TIL. Generally, these cells expressed higher levels of PD-1 and, defined as PD-1 high (PD-1^hi^), which likely signals that these cells are either dysfunctional [69] or follicular helper T cells (Tfh) [70]. LAG3 gene expression by microarray data was associated with positive clinical outcomes in basal-like BC (~80% of triple negative BC) and was also frequently expressed with other immune checkpoint molecules such as TIM3. Several contemporaneous BC studies investigated LAG3 expression using immunohistochemistry (IHC) to correlate its presence with prognosis. In one study, intratumoral LAG3^+^ TIL were detected in 11% of tumors and significantly associated with more aggressive clinicopathological parameters, including: Young age, large tumor size, high proliferation, which also typically characterize the more aggressive HER2-enriched and basal-like BC subtypes [70]. Multivariate analyses identified BC patients with intratumoral LAG3^+^ TIL as having significantly improved BC specific survival (BCSS) (hazard ratio (HR): 0.71, 95% confidence interval (CI) 0.56–0.90), particularly if they were ER-negative (HR: 0.50, 95% CI 0.36–0.69). LAG3 on HER2-enriched and basal-like BC signifies that these patients have a good prognosis despite their diagnosis with an aggressive BC subtype. Interestingly, around half of PD-L1^+^ and two-thirds of PD-1^+^ tumors were also positive for intratumoral LAG3^+^ TIL in this study. Concurrent LAG3^+^ and CD8^+^ intratumoral TIL was also associated with increased BCSS (HR: 0.49, 95% CI 0.32–0.74). These data highlight the notion that not only LAG3 expression but the location of TIL expressing this checkpoint molecule can have a positive impact on clinical outcome in these aggressive BC subtypes.

PD-1 and LAG3 were shown to be concomitantly expressed in approximately 15% of TNBC patients and positively correlated with a TIL presence in another study [71]. However, PD-1 and LAG3 expression was not significantly associated with patient outcome in this dataset, potentially because authors did not perform spatial analysis of LAG3^+^ cells in the TME [72]. Their data identified an increased probability of achieving a pathologic complete response following neoadjuvant treatment if pre-treatment biopsies highly expressed LAG3, finding it was correlated with PD-1 and PD-L1 expression. Alternatively, in a recent study, patients with residual disease after neoadjuvant chemotherapy that were characterized by high LAG3 expression, and in particular associated with PD-L1 expression, had a poor prognosis [71]. Overall, these studies show that LAG3 expression in BC is associated with extensive immune infiltration and frequently associated with other immune checkpoint molecules (particularly PD-1/PD-L1), providing a strong rationale for targeting both LAG3 and PD-1/PD-L1 concurrently or sequentially. This idea is supported by the finding of LAG3 upregulation on TIL in MHC class II^+^ tumors that develop resistance to anti-PD-1 agents [73]. Some immune checkpoint molecules like TIGIT that are less expressed in breast tumors, which differs from CTLA-4, PD-1 and TIM3 [74] whose genes are upregulated, thus resulting more expressed in BC. On the contrary one study found that TIGIT was more frequently expressed than other the ligand for PD-1, PD-L1, in in situ lesions of the breast. Instead PD-L1 was more frequently detected in TN invasive tumors [75], signifying that the pattern of expression of these immune checkpoint molecules might vary in different phases of the disease (from in situ to invasive). In another study, peripheral blood from BC and colorectal cancer patients, observed LAG3 downregulation in patients with TIM3, TIGIT and PD-L1 upregulation [76], confirming the heterogeneous expression of these molecules also in circulating immune cells, and contributing to the complexity of this scenario.

## 5. LAG3 Blockade in Cancer and Clinical Trials Testing LAG3 Targeting Compounds

**Figure 3:**
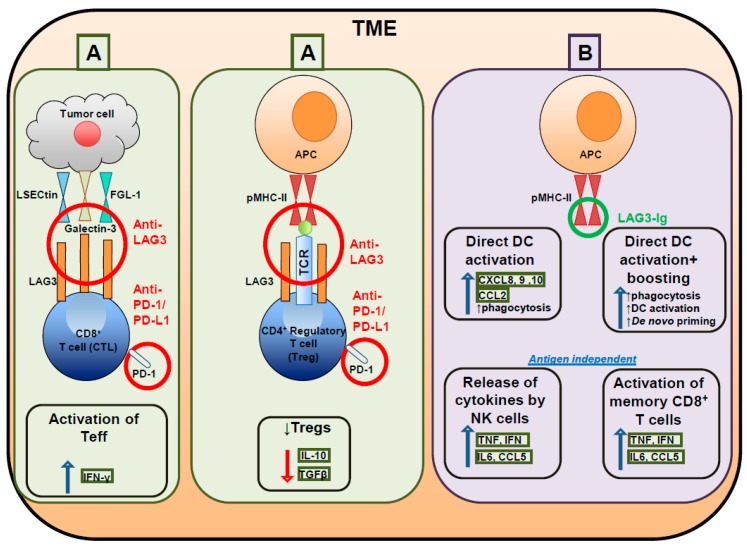
Targeting effector and regulatory T cells with LAG3 antagonistic antibodies (**A**) and activating antigen presenting cells with soluble LAG3 Immunoglobulin (Ig) (**B**). (**A**) Binding of LAG3 antagonistic antibodies (Abs) to LAG3 prevents its interaction with the respective ligands (LSECtin, galectin-3 and FGL-1 for CD8^+^ T cells; antigen presenting cells (APC) for CD4^+^ T cells). This contributes to releasing the break to the activation of tumor-infiltrating lymphocytes (TIL) and is achieved with the use of a combined blockade with anti-PD-1/PD-L1 Abs. (**B**) Binding of a soluble LAG3 Ig to APC engages MHC-II molecules on the cell surface. This generates an upregulation of co-stimulatory molecules, such as CD80, CD86 and CD40, and leads to the secretion of pro-inflammatory cytokines and chemokines by APC. APC activation through soluble LAG3 agonistic Ab may induce a rapid release of pro-inflammatory cytokines by a subpopulation of NK cells and activation of memory CD8^+^ T cells in an antigen independent manner. APC: Antigen presenting cell; CCL: chemokine (C-C motif) ligand; CXCL: chemokine (C-X-C motif) ligand; DC: dendritic cell; FGL-1: fibrinoigen like protein - 1; IFN: interferon; Ig: immunoglobulin; IL: interleukin; LSECtin: liver and lymph node sinusoidal endothelial cell C-type lectin; NK: natural killer; PD-1: programmed cell death -1; Teff: effector T cells; TNF: tumor necrosis factor; Tregs: regulatory T cells.

LAG3 regulates the proliferation, cytokine production and/or cytolytic functions of T cells through its cytoplasmic domain. Although most of the molecular mechanisms remain poorly understood (Figure 2), it is known that LAG3 recognition of stable peptide-MHC-II complexes is critical for activity [38]. Consequently, LAG3 expression signals that ongoing responses are active at the inflammatory site (i.e. the TME). The rationale for targeting LAG3 is based on the presence of a pre-existing immune response. Peptide-MHC-II complexes are generated following activation-induced by IFNγ release and LAG3 expression is dependent upon activation [77], providing the logic for clinically targeting LAG3. Blocking LAG3 has been shown to be active in association with anti-PD-1 or anti-PD-L1 agents in cancer patients. The main consequences of dual inhibition are: 1) Inhibiting Treg activities, 2) promoting DC maturation, and 3) rescuing dysfunctional CD4^+^ Th TIL and CD8^+^ CTL TIL (Figure 3).

The selection of LAG3 as partner with an anti-PD-1 or anti-PD-L1 blocking Ab has a hypothetical advantage with respect to CTLA-4 because of its lower toxicity. This however remains to be demonstrated in further studies and future clinical trials designed to compare these targets (i.e., CTLA-4 versus LAG3) as partners for anti-PD-1 or anti-PD-L1 agents. LAG3 is now being tested in clinical trials of patients previously treated with anti-PD-1/PD-L1 agents and preliminary data from melanoma patients has shown efficacy of this dual blockade [78]. Alternatively, CTLA-4 combined with the anti-PD-1 nivolumab given to immunotherapy naïve patients shows higher activity and efficacy coupled with a higher incidence rate of irAEs [79].

There are currently several agents targeting LAG3 that are being tested in clinical trials (summarized in Table 1). The earliest trial employed a soluble dimeric recombinant protein that contained four LAG3 extracellular domains in a soluble LAG3-immunoglobulin (Ig) named IMP321 [80]. This compound was initially designed to be a LAG3 antagonist, but it is currently being used as an immune adjuvant to activate APC because it binds with high affinity to MHC-II expressed on DC that mature and migrate to lymph nodes [81]. Early studies found that when the LAG3-Ig interacted with MHC-II on immature DC it induced CD80/86 upregulation, IL2 and TNFα secretion and promoted morphological changes in DC [82]. Its adjuvant function is to enhance cross presentation of Ag to T cells and thereby activate CD8^+^ T cell responses. IMP321, tested as a monotherapy (NCT00351949) or in combination with chemotherapy (NCT00732082 and NCT00349934) or vaccines [81], showed minimal activity in monotherapy [83] and modest activity using combination approaches [84].

Early data from the anti-LAG3 relatlimab used as simultaneous dual blockade targeting PD-1 together with LAG3 produced an objective response rate of 11% in melanoma patients progressing after anti-PD-1/PD-L1 monotherapy [85]. Translational data suggest that responses were associated with LAG3 expression in the TME, indicating that LAG3 might constitute a biomarker for patient selection. Relatlimab is currently being tested in microsatellite stable advanced colorectal carcinoma (NCT03642067), mismatch repair-deficient tumors resistant to prior PD-1/PD-L1 inhibition (NCT03607890), advanced chordoma (NCT03623854), advanced melanoma (NCT03470922, NCT02519322), gastro/esophageal cancer (NCT03044613, NCT03610711, NCT02935634) or gastroesophageal junction (NCT03662659), multiple solid cancers (NCT03459222, NCT01968109, NCT02966548, NCT03335540), advanced renal cell carcinoma (NCT02996110), virus-associated tumors (NCT02488759), non-small cell lung cancer (NCT02750514), hematological malignancies (NCT02061761) and glioblastoma (NCT03493932).

LAG3 antagonists have also been produced and are currently being tested in immunotherapy trials as a co-drug for simultaneously blocking the PD-1/PD-L1 pathway. The rationale for these trials is derived from preclinical data supporting its synergy with PD-1 blockade [86]. Bispecific Abs targeting LAG3 with Fc-domains for Ag binding to LAG3 were capable of binding human and murine LAG3, inhibiting its interaction with MHC-II and inducing IL-2 production in a T cell assay [28]. LAG3 is being targeted together with PD-1 in the dual immunomodulator MGD013 (ClinicalTrials.gov identifier: NCT03219268, phase I trial) whose objective is to inhibit both targets and induce a synergistic effect on tumor immunity, as previously shown in murine models [86]. Other drugs interacting with LAG3 include: LAG525, REGN3767, TSR-033 and GSK2831781 [87]. LAG525 is being tested for hematological malignancies (NCT03365791), TNBC (NCT03499899), advanced malignancies (NCT02460224) and metastatic melanoma (NCT03484923). REGN3767 is being investigated for advanced cancers (NCT03005782), TSR-033 in advanced solid tumors (NCT03250832) and GSK2831781 for psoriasis (NCT02195349).

## 6. Conclusions

The results from the various LAG3 clinical studies are currently unknown but the rationale behind them is based on data suggesting that co-targeting LAG3 is a promising approach for improving immunotherapy responses in multiple human tumor types. LAG3 co-expression with other immune checkpoint molecules, including PD-1, PD-L1 and TIM3, has been documented indicating that combinatorial immunotherapies targeting multiple TME immunosuppressive pathways hold promise. However, safety data must first demonstrate that simultaneous or sequential combinations are feasible and tolerable.

## Figures and Tables

**Table 1:** Ongoing clinical trials using LAG3 blockade to treat cancer patients.

Reference	Drug(s)	Phase	Tumor Type	Main Objectives	Status
NCT 02676869	IMP321 (LAG3 Ig fusion protein-eftilagimod alpha) + pembrolizumab (anti-PD-1)	I	Stage IV and stage III melanoma	Safety, tolerability and recommended phase 2 dose	Active, not yet recruiting
NCT 02614833	IMP321 (LAG3 Ig fusion protein-eftilagimod alpha) + paclitaxel	IIb, randomized	Hormone receptor-positive stage IV breast cancer	Recommended phase II dose, safety and efficacy (survival and objective response rate)	Recruiting
NCT 03252938	IMP321 (LAG3 Ig fusion protein-eftilagimod alpha)	I	Metastatic solid tumors; peritoneal carcinomatosis	Feasibility and safety of intra-tumoral and intra-peritoneal (new routes of administration), and subcutaneous injections	Recruiting
NCT 03625323	IMP321 (LAG3 Ig fusion protein-eftilagimod alpha) + pembrolizumab (anti-PD-1)	II	Advanced or metastatic non-small cell lung carcinoma and head and neck squamous cell carcinoma	Safety and efficacy (objective response rate)	Recruiting
NCT 03642067	Relatlimab (anti-LAG3 monoclonal antibody BMS-986016) + nivolumab (anti-PD-1)	II	Microsatellite stable (MSS) colorectal carcinomas Colorectal carcinoma	Response (objective response rate) and biomarkers	Recruiting
NCT 03623854	Relatlimab (anti-LAG3 monoclonal antibody BMS-986016) + nivolumab (anti-PD-1)	II	Advanced chordomas	Clinical benefits (objective response rate, progression free survival) and safety	Not yet recruiting
NCT 03743766	Relatlimab (anti-LAG3 monoclonal antibody BMS-986016) +/- nivolumab (anti-PD-1)	II	Stage III or stage IV melanoma	Change in LAG3 and PD-1 expression; change in tumor size and overall response rate	Recruiting
NCT 03607890	Relatlimab (anti-LAG3 monoclonal antibody BMS-986016) + nivolumab (anti-PD-1)	II	Refractory microsatellite unstable high (MSI-H) solid tumors prior of PD-(L) 1 therapy MSI-H tumors	Objective response rate, toxicity, survival, disease control rate, best overall response, duration of response, duration of clinical benefit, time to objective response	Recruiting
NCT 03724968	Relatlimab (anti-LAG3 monoclonal antibody BMS-986016) +/- nivolumab (anti-PD-1); nivolumab + ipilimumab (anti-CTLA-4)	II	Stage III and stage IV melanoma, stratified by MHC-II expression	Efficacy, measured by change in activated GZMB+ CD8+ T-cell density intratumorally; response rate, median progression free survival, overall survival, and safety and tolerability of nivolumab plus relatlimab in patients with MHC-II (+) melanoma, and of nivolumab plus ipilimumab in patients with MHC-II (-) melanoma; explore potential associations of biomarkers with clinical efficacy and/or incidence of adverse events due to study drug by analyzing biomarker measures within the peripheral blood and tumor microenvironment	Recruiting
NCT 03470922	Nivolumab (anti-PD-1) +/- relatlimab (anti-LAG3 monoclonal antibody BMS-986016)	II/III	Previously untreated stage III or stage IV melanoma	Survival (progression free survival, overall survival), responses (objective response rate), duration of response, toxicity	Active, not yet recruiting
NCT 03044613	Nivolumab (anti-PD-1) or Nivolumab plus relatlimab (anti-LAG3 monoclonal antibody BMS-986016); chemoradiation	Ib	Stage II/III gastric cancer, esophageal cancer, gastroesophageal cancer	Toxicity, feasibility, pathologic complete response rate; approximate quantitation of infused nivolumab bound to PD-1 receptors on the surface of T cells in the peripheral blood and within the resected tumor and lymph node specimens; changes in expression of selected immune markers; survival (overall and recurrence free survival)	Recruiting
NCT 03459222	Relatlimab (anti-LAG3 monoclonal antibody BMS-986016) + nivolumab (anti-PD-1) or + BMS-986205 (IDO1 inhibitor) or relatlimab + nivolumab and ipilimumab (anti-CTLA-4)	I/II	Advanced malignant tumors	Toxicity, safety, objective response rate, disease control rate, median duration of response	Recruiting
NCT 03867799	Relatlimab (anti-LAG3 monoclonal antibody BMS-986016) + nivolumab (anti-PD-1)	II	Metastatic colorectal cancer	Disease control rate, toxicity, duration of disease control, best objective response rate, progression free survival, overall survival	Not yet recruiting
NCT 02519322	Nivolumab (anti-PD-1); nivolumab + relatlimab (anti-LAG3 monoclonal antibody BMS-986016); nivolumab + ipilimumab (anti-CTLA-4)	II	Melanoma	Pathologic response; immunological response (changes in T cell infiltrate); objective response; survival (recurrence free and overall survival); adverse events	Recruiting
NCT 03704077	Relatlimab (anti-LAG3 monoclonal antibody BMS-986016) + nivolumab (anti-PD-1) + paclitaxel; nivolumab + paclitaxel; ramucirumab + paclitaxel; relatlimab + nivolumab; nivolumab	II	Recurrent, locally advanced, or metastatic gastric cancer (GC) or gastroesophageal junction (GEJ) adenocarcinoma	Overall response rate; toxicity; duration of response; survival (progression free survival and overall survival)	Not yet recruiting
NCT 01968109	Relatlimab (anti-LAG3 monoclonal antibody BMS-986016) + nivolumab (anti-PD-1)	I/II	Advanced solid tumors	Safety (adverse events), tolerability and efficacy (objective response rate, disease control rate, duration of response)	Recruiting
NCT 03662659	Relatlimab (anti-LAG3 monoclonal antibody BMS-986016) + investigator’s choice chemotherapy; nivolumab (anti-PD-1) + investigator’s choice chemotherapy	II	Unresectable, untreated, locally advanced or metastatic gastric or gastroesophageal junction cancer	Objective response rate; toxicity; duration of response; survival (overall survival and progression free survival)	Recruiting
NCT 03610711	Nivolumab (anti-PD-1) +/- relatlimab (anti-LAG3 monoclonal antibody BMS-986016) + stereotactic radiotherapy	Ib/II	Recurrent or limited metastatic gastroesophageal cancer	Change in the infiltrating CD8+ T cell density; safety; efficacy	Recruiting
NCT 02966548	Relatlimab (anti-LAG3 monoclonal antibody BMS-986016) +/- nivolumab (anti-PD-1)	I	Advanced solid tumors	Safety, tolerability, and efficacy	Recruiting
NCT 02996110	Relatlimab (anti-LAG3 monoclonal antibody BMS-986016) + nivolumab (anti-PD-1); nivolumab + BMS-986205 (IDO-inhibitor); nivolumab + ipilimumab (anti-CTLA-4)	II	Advanced renal cell carcinoma	Objective response rate; duration of response; progression free survival rate; safety; tolerability	Recruiting
NCT 02935634	Relatlimab (anti-LAG3 monoclonal antibody BMS-986016) + nivolumab (anti-PD-1); nivolumab + ipilimumab (anti-CTLA-4); nivolumab and BMS-986205 (IDO-inhibitor)	II	Advanced gastric cancer	Objective response rate; duration of response; progression free survival rate; adverse events	Recruiting
NCT 02488759	Nivolumab (anti-PD-1); nivolumab + ipilimumab (anti-CTLA-4); nivolumab + relatlimab (anti-LAG3 monoclonal antibody BMS-986016); nivolumab + daratumumab (anti-CD38)	I/II	Virus associated tumors (anal canal cancer; cervical cancer; Epstein Barr Virus (EBV) positive gastric cancer; HPV positive and negative squamous cell cancer of the head and neck (SCCHN); Merkel cell cancer; nasopharyngeal cancer; penile cancer; vaginal and vulvar cancer	Safety, tolerability; objective response rate; survival (progression free survival; overall survival); duration of response	Recruiting
NCT 02750514	Nivolumab (anti-PD-1); nivolumab + dasatinib (src, c-Kit, ephri receptor inhibitor); nivolumab + relatlimab (anti-LAG3 monoclonal antibody BMS-986016); nivolumab + ipilimumab (anti-CTLA-4); nivolumab + BMS-986205 (IDO-inhibitor)	II	Advanced non-small cell lung cancer	Objective response; duration of response; progression free survival rate; safety and tolerability	Recruiting
NCT 03335540	Nivolumab (anti-PD-1) + relatlimab (anti-LAG3 monoclonal antibody BMS-986016); nivolumab + lirilumab (anti-KIR monoclonal antibody); nivolumab + cabiralizumab (anti-CSF1R); nivolumab + ipilimumab (anti-CTLA-4); nivolumab + anti-GITR; nivolumab + BMS-986205 (IDO-inhibitor); nivolumab + radiation therapy	I	Solid tumors	Number of patients with biopsy specimens; change from baseline in histopatologic and biomarker expression patterns; number of adverse events;	Recruiting
NCT 03219268	MGD013 (Anti-PD-1, anti-LAG-3 bispecific DART protein)	I	Unresectable, locally advanced or metastatic solid tumors of any histology	Adverse events; pharmacokinetics; pharmacodynamics; immunogenicity, and preliminary antitumor activity	Recruiting
NCT 03365791	LAG525 (anti-LAG3 IgG4 antibody) + PDR001 (anti-PD-1 IgG4 antibody)	II	Advanced solid and hematological malignancies	Clinical benefit rate; progression free survival; overall response rate; time to response; safety and tolerability; duration of response; time to progression	Active, not recruiting
NCT 03499899	LAG525 (anti-LAG3 IgG4 antibody) + PDR001 (anti-PD-1 IgG4 antibody); LAG525 + PDR001 + carboplatin; LAG525 + carboplatin	II	Advanced triple negative breast cancer (first or second line)	Overall response rate; duration of response; overall survival; pharmacokinetic parameters; time to response; progression free survival; clinical benefit rate; anti-drug antibodies prevalence at baseline and incidence on treatment	Recruiting
NCT 02460224	LAG525 (anti-LAG3 IgG4 antibody) +/- PDR001 (anti-PD-1 IgG4 antibody)	I/II	Advances malignancies	Dose limiting toxicities; overall response rate; area under the curve; concentration of anti-LAG525 and anti-PDR001 antibodies; correlation of PD-L1 and LAG-3 expression; overall response rate; expression of IFN-γ immune-related genes by mRNA profiling; safety; tolerability; progression free survival; duration of response; disease control rate	Active, not recruiting
NCT 03742349	LAG525 (anti-LAG3 IgG4 antibody) + PDR001 (anti-PD-1 IgG4 antibody) + NIR178 (adenosine A2A receptor antagonist); LAG525 + PDR001 + capmatinib (c-Met inhibitor); LAG525 + PDR001 + MCS110 (anti-M-CSF antibody); LAG525 + PDR001 + canakinumab (anti-interleukin-1 beta)	Ib	Adult patients with triple negative breast cancer	Safety; dose limiting toxicities; best overall response; progression free survival; presence of anti-PDR001. of anti-LAG525, of anti-MCS110, of anti- canakinumab antibodies; pharmacokinetic and pharmacodynamic parameters	Recruiting
NCT 03484923	LAG525 (anti-LAG3 IgG4 antibody) + PDR001 (anti-PD-1 IgG4 antibody); PDR001 + capmatinib (c-Met inhibitor); PDR001 + canakinumab (anti-interleukin-1 beta)	II	Previously treated stage III and stage IV melanoma	Overall response rate; duration of response; survival (overall survival and progression free survival); disease control rate; prevalence and incidence of anti-drug antibodies; frequency of patients with a favorable biomarker profile	Recruiting
NCT 03005782	REGN3767 (anti-LAG3 monoclonal antibody) +/- REGN2810 (anti-PD-1 monoclonal antibody)	I	Advanced tumors	Safety; tolerability; activity and pharmacokinetics	Recruiting
NCT 03250832	TSR-033 (humanized monoclonal anti-LAG3 IgG4) +/- anti-PD-1 (IgG4 antibody)	I	Advanced solid tumors	Adverse events; responses; pharmacokinetic parameters; duration of response; disease control rate; survival (progression free survival; overall survival)	Recruiting
